# Perspective on Data‐Sharing Requirements for the Necessary Evolution of Drug Development

**DOI:** 10.1002/jcph.1607

**Published:** 2020-03-28

**Authors:** Jeffrey S. Barrett

**Affiliations:** ^1^ Bill & Melinda Gates Medical Research Institute Cambridge Massachusetts USA

**Keywords:** data sharing, honest broker, open data, collaboration

At the 2019 annual meeting of the American College of Clinical Pharmacology (Chicago) one of the plenary sessions discussed the expanding roles of data sharing and collaboration. The predominant benefit of data sharing and collaboration is accelerated scientific progress. Advances are clearly valuable to both the pharmaceutical industry and academic researchers in addition to clinicians and the entire health care system, especially when translated into improved patient outcomes, reduced research costs, and decreased time in moving discoveries from the bench to the bedside. Despite the anticipated benefits, sharing research data is still viewed as a work in progress. Likewise, short‐term costs to provide data‐sharing services are likely to be an issue for some. There were a few obvious take‐home messages that resonated from the session. Within the pharmaceutical industry, most real sharing is still occurring in the precompetitive space or targeted in populations in which the financial gains are modest or nonexistent (eg, pediatric oncology, rare diseases, and global health settings). Academic – industry collaborations are broad and can be difficult based on intellectual property considerations and other incentivization issues. Contract research organizations and others in the technology sector will also need to play an important role to bring forward solutions for diverse stakeholders. Meaningful collaboration still requires mutual understanding, and sharing is still problematic for a variety of reasons.

Some positive examples exist, but sustainability is seemingly always in question. Noticeable examples include the National Heart, Lung, and Blood Institute's biological specimen and data repository, Project Data Sphere and the Project Genomics Evidence Neoplasia Information Exchange of the American Association for Cancer Research.^1^, ^2^ Althogh most of these are predominantly academic examples, there are diverse constituency examples as well. Barriers are typically not an issue of technology limitations.^3^ Some of the bottlenecks include the value/overvalue of intellectual property, a lack of resources and expertise, the lack of sharing history or culture of sharing, lack of data‐sharing policies and implementation, and the lack of trust for governance around sharing. To be fair the overvalue of intellectual property sentiment is often a reflection of the conservative low‐risk legal perspective that typically dictates data‐sharing decisions. Clearly, it is in the best interest of the company to protect itself from legitimate intellectual property infringement given the increasingly competitive landscape, but this should not come at the expense of the gains (scientific and financial) that could be made from legitimate collaboration. Some potential solutions have been proposed, but these have mostly been implemented for academic collaborations,^4^, ^5^ and industry has been slow to adopt more open data sharing within their organizations, preferring a more traditional Biostat/data management governance model enforced by standard operating procedures focused on protecting clinical data.

Although issues such as data deidentification and the potential for unauthorized reidentification have prevented some from considering sharing and even collaborating, progress has been made in these areas. Analyses of distributed data sets require technical infrastructure and funding to support and maintain the compute environment. It should be possible to achieve meaningful data sharing with embedded research that encourages rather than discourages the growth of a learning health system.^6^ Although there is often a mismatch between the explicit motivations, unstated or implicit motivations, and the design of an actual data‐sharing policy, this should not dissuade us from pursuing such agreements.^7^ As has been observed already, the shift from an aim of changing behavior to changing culture has both subtle and profound implications for policy design and implementation.^8^

One potential solution to the issue of data‐sharing governance is an honest broker approach. An honest broker is an entity that keeps sets of private information but distributes parts of those sets to other entities who should not have access to the entire set. The honest broker approach for data sharing offloads the burden of housing identifiable data elements of protected health information (eg, name and address) as well as manage data transfer between clinical and research systems.^3^, ^4^, ^9^ The benefit to the sponsor, of course, is the ability to outsource the logging of requests and distribution of data based on contractual rules of engagement tied to data sensitivity. Another advantage is that internal company resources are not used for these activities. Although this is typically a fee‐for service activity for the partner organization, there are some differences from contract to contract, based on the data source and type. Two of the more public examples include the Yale Open Data Access project,^10^ https://yoda.yale.edu/, and the Duke Clinical Research Institute, https://dcri.org/our-work/analytics-and-data-science/data-sharing/,^11^ which serve in such a capacity for Johnson & Johnson and Bristol Meyers Squib pharmaceuticals, respectively. Although these represent a step in the right direction, sharing is still based on low‐risk postapproval data only. Likewise, even though honest broker approaches have been engaged for some time, there are still questions about access polices and costs associated with the service as well as potential biases to grantees based on such access.^12^ The details of these arrangements, particularly around liability and other legal implications, are also not completely known, and the honest broker as a model is hardly at a stage inwhich we could consider best practices. Likewise, the honest broker is not the only viable approach, and other third‐party solutions involving contract research organizations are also available though not necessarily inexpensive alternatives. A problem with the honest broker approach is that they become a target for hackers and malicious insiders, of course.

Other recommendations for improving sharing within the context of drug development include necessary improvements to the manner and mechanism of internal pharma‐sharing solutions. Specifically, these environments need to be more in line with a federated governance model (governance balanced between a central authority and constituent units) and based on information technology (IT) solutions that permit more flexible sharing rules accommodating complex sharing with diverse internal partners, improved and broader data‐sharing agreements reflecting the intellectual property considerations of diverse external stakeholders and sharing considerations that can change over time (eg, new partners, change in partners, revised agreements). The generation of an honest broker approach best practices and other commercial solutions that reflect diverse stakeholders and accommodate global data privacy concerns would also help. This does not preclude sponsors from homegrown solutions, of course, although the time and expense including the loss of head count to these activities are not trivial.

Since 2014 the industry has endorsed a commitment to share deidentified individual patient data on request.^13^ Two separate studies have confirmed that the extent to which this actually occurs within a reasonable time frame (2 years) is 15% or less.^14^, ^15^ Issues identified were highlighted by the lack of data‐sharing policies/processes and data‐sharing policy conditions that exclude access on the basis of ongoing follow‐up and regulatory activity. Although implementation undoubtedly takes time, it would seem that progress is stalled at the moment. Data sharing is in fact a well‐studied problem, and other industries (eg, publishing and financial) have worked through their own growing pains to engage in more meaningful sharing. For the pharmaceutical industry to sustain itself and embrace the innovation and collaboration necessary to thrive in a value‐based health care system, it will have to learn to share in a manner it is unaccustomed to and address any IT and legal barriers in addition to adopting the requisite internal policies. Likewise, academic investigators will need to cope with internal intellectual property concerns and embrace potential stewardship in an open manner and do their part to address the requisite guidelines, standard operating procedures, and governance to ensure that they are appropriate sharing partners. This will require a more transparent conversation with all relevant stakeholders in which the benefit:risk to sharing is objectively calibrated.
